# Hepatic Stellate Cell Activation and Inactivation in NASH-Fibrosis—Roles as Putative Treatment Targets?

**DOI:** 10.3390/biomedicines9040365

**Published:** 2021-03-31

**Authors:** Alexandra Zisser, David H. Ipsen, Pernille Tveden-Nyborg

**Affiliations:** 1 Department of Veterinary and Animal Sciences, Faculty of Health and Medical Sciences, University of Copenhagen, Ridebanevej 9, 1870 Frederiksberg C, Denmark; alexandra.zisser@sund.ku.dk; 2 Liver Disease Research, Novo Nordisk A/S, Novo Nordisk Park 1, 2760 Måløv, Denmark; DVIE@novonordisk.com

**Keywords:** non-alcoholic fatty liver disease, non-alcoholic steatohepatitis, fibrosis, hepatic stellate cells, HSC activation, HSC inactivation

## Abstract

Hepatic fibrosis is the primary predictor of mortality in patients with non-alcoholic steatohepatitis (NASH). In this process, the activated hepatic stellate cells (HSCs) constitute the principal cells responsible for the deposition of a fibrous extracellular matrix, thereby driving the hepatic scarring. HSC activation, migration, and proliferation are controlled by a complex signaling network involving growth factors, lipotoxicity, inflammation, and cellular stress. Conversely, the clearance of activated HSCs is a prerequisite for the resolution of the extracellular fibrosis. Hence, pathways regulating the fate of the HSCs may represent attractive therapeutic targets for the treatment and prevention of NASH-associated hepatic fibrosis. However, the development of anti-fibrotic drugs for NASH patients has not yet resulted in clinically approved therapeutics, underscoring the complex biology and challenges involved when targeting the intricate cellular signaling mechanisms. This narrative review investigated the mechanisms of activation and inactivation of HSCs with a focus on NASH-associated hepatic fibrosis. Presenting an updated overview, this review highlights key cellular pathways with potential value for the development of future treatment modalities.

## 1. Introduction

Around a quarter of the world’s adult population are predicted to have non-alcoholic fatty liver disease (NAFLD) [1]. An estimated 7–30% of these patients develop non-alcoholic steatohepatitis (NASH), characterized by progressing inflammation and fibrosis that compromise liver function and patient health [1,2]. Hepatic fibrosis constitutes a primary predictor of mortality and adverse liver events in NAFLD patients, and it is caused by the activation of liver resident myofibroblasts that primarily consist of hepatic stellate cells (HSCs) and a smaller population of portal myofibroblasts, resulting in the subsequent deposition of a fibrous extracellular matrix (ECM) central to NASH-related fibrosis [3,4,5,6]. Just as HSC activation is a key event in the development and progression of hepatic fibrosis, the elimination of activated HSCs (aHSCs) is pivotal for fibrosis resolution [7]. In this way, HSCs represent an attractive therapeutic target against advanced NASH; however, the cellular mechanisms underlying HSC activation and elimination in the liver remain to be fully elucidated [8,9]. This narrative review provides an update on the mechanisms involved in the regulation of HSCs and their putative role in the current treatment modalities of NASH-related fibrosis.

## 2. NAFLD Etiology and the Role of Hepatic Stellate Cells

**Figure 1:**
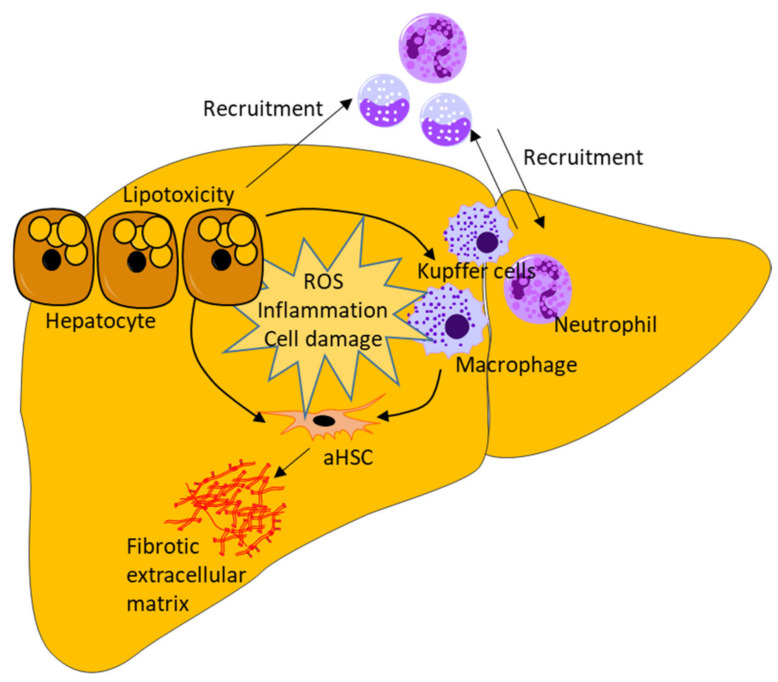
A simplified overview of primary drivers of non-alcoholic steatohepatitis (NASH)-induced hepatic fibrosis. The excessive accumulation of triglycerides and free fatty acids increases hepatocellular lipid oxidation generating reactive oxygen species (ROS) and lipotoxicity. This leads to cellular damage and the release of inflammatory cytokines, prompting the activation of resident liver macrophages (Kupffer cells) and the recruitment of circulating immune cells: monocytes and leukocytes. The initial hepatic steatosis then becomes a state of hepatic inflammation and progresses to NASH. The inflammation and sustained lipotoxicity maintain a self-perpetuating vicious circle of increased production of ROS, inflammation, and cell damage, ultimately promoting the activation of hepatic stellate cells (aHSC), which leads to the formation of a fibrogenic extracellular matrix, thus hallmarking the transition to a state of NASH-induced fibrosis.

NAFLD defines a spectrum of liver diseases characterized by hepatic steatosis not due to excessive alcohol consumption [10]. Multiple factors are involved NAFLD development and progression; however, dyslipidemia is a central feature in most patients. An excessive intake of energy in the form of fat and carbohydrates results in the hepatic accumulation of lipids and lipotoxicity [11]. Continued fatty acid oxidation generates increased levels of reactive oxidant species and cytotoxic lipid metabolites, which promote oxidative and lipotoxic stress that lead to cellular damage and inflammation, hallmarking the progression from simple steatosis to NASH (defined by hepatic steatosis, inflammation, and the presence of ballooning hepatocytes) [11,12]. Infiltrating immune cells, together with liver-resident macrophages (Kupffer cells), secrete pro-inflammatory and -fibrotic cytokines that drive the inflammation and create a self-propagating vicious circle of hepatocellular stress and damage [7,13] (a brief, schematic overview of general mechanisms is shown in Figure 1). The crosstalk between inflammation, growth factors, nuclear receptor signaling, ECM interactions, and metabolic signals promotes the activation of HSCs and portal myofibroblasts, leading to hepatic scarring/fibrosis and ultimately compromising hepatic function, as described in detail later in the manuscript [3,14]. This activation induces the production of fibrous collagens and stimulates the proliferation and migration of HSCs and portal myofibroblasts, thus allowing for the advancement of the fibrous ECM. The fibrosis of the hepatic parenchyma commonly begins perivenularly in zone 3 (stage F1), progresses to portal and periportal areas (stage F2), and can advance to bridging fibrosis (stage F3) and cirrhosis (stage F4), ultimately showing severe structural changes in liver morphology and deviated angiogenesis [15,16]. In addition to HSC activation, myofibroblasts residing in the portal area are activated to produce a fibrous ECM. In this regard, the portal myofibroblasts resemble HSCs but do not express the same surface markers or carry vitamin A droplets [17,18]. Portal myofibroblasts are situated around the bile ducts, and the concurrent deposition of a fibrotic ECM is linked to cholangiocytes (bile duct epithelia) and fibrosis of the biliary system, e.g., cholestatic fibrosis, also reported in NASH [6,18]. Hepatic fibrosis increases all-cause mortality, liver-related mortality, and the risk of liver transplantation in patients with NASH [4].

**Figure 2:**
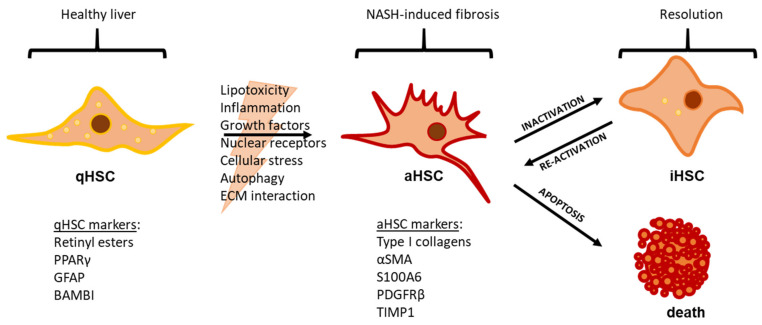
The hepatic stellate cell phenotypic switch in NASH. In a healthy liver, the hepatic stellate cell (HSC) rests in a quiescent state (qHSC) while residing close to the hepatic sinusoids. qHSCs are considered dormant and non-proliferative, and they are characterized by the cytoplasmatic storage of retinyl esters (vitamin A) in lipid droplets; markers include PPARγ, GFAP, and BAMBI, all expressed in the qHSCs. The accumulation of lipotoxic metabolites, inflammation, and oxidative stress in NASH affects multiple hepatic cell types and leads to the release/activation of several cellular signaling factors, such as growth factors (e.g., increased TGFβ, PDGF, and connective tissue growth factors) and nuclear receptors (e.g., decreased PPARγ and retinoid X receptor activation), thus promoting an HSC phenotypic switch. In this process, qHSCs lose their stored retinyl esters and transdifferentiate into the activated, proliferative, and contractile state (aHSC). aHSCs are characterized by the production of pro-collagens for extracellular matrix deposition and the promotion of HSC activation and fibrogenesis (thus creating a positive feedback loop), as well as the ability to migrate and divide; markers include the expression of αSMA, S100a6, PDGFRβ, and TIMP1. The clearance of aHSCs is necessary for the cessation of matrix deposition, and it can take place through apoptosis or through inactivation. Inactivated HSCs (iHSCs) differentiate towards a more dormant phenotype (e.g., with a decrease of aHSC characteristics and the re-establishment of the cytoplasmic storage of retinyl esters), but they do not completely revert to the qHSC state and have increased sensitivity toward reactivation. aHSC: activated hepatic stellate cell; BAMBI: bone morphogenetic protein and activin membrane bound inhibitor; ECM: extracellular matrix; GFAP: glial fibrillary acidic protein; iHSC: inactivated hepatic stellate cell; PDGFRβ: platelet derived growth factor receptor β; PPARγ: peroxisome proliferator activated receptor γ; qHSC: quiescent hepatic stellate cell; S100a6: S100 calcium-binding protein A6; TGFβ: transforming growth factor beta; TIMP1: tissue inhibitor of metalloproteinase 1; αSMA: alpha smooth muscle actin.

HSCs account for roughly 10% of all liver cells and reside in the space of Disse (the perisinusoidal space), lying between hepatocytes and with cellular extensions surrounding the sinusoidal endothelium that maintain consistent exposure to hepatic blood flow [19]. In their dormant state, HSCs display a quiescent, non-proliferative phenotype (qHSCs) and are characterized by storing retinyl esters (vitamin A), cholesteryl esters, and triglycerides in cytosolic lipid vacuoles [20,21]. qHSCs are thought to contribute to ECM homeostasis, hepatocyte proliferation, innate immunity, and sinusoidal blood flow regulation [22,23]. Upon liver injury, qHSCs become activated and transdifferentiate into aHSCs (myofibroblasts), losing their lipid storage droplets and exhibiting a contractile, proliferative, and fibrogenic phenotype, together with vast changes in the gene expression profile [24,25,26,27] (Figure 2).

The contractile activity of aHSCs is characterized by the expression of alpha smooth muscle actin (αSMA; encoded by *Acta2*) and S100a6 (S100 calcium-binding protein A6), the formation of stress fibers, and the deposition of ECM components [28]. Fibrillary collagens (e.g., collagen type I, which is encoded by *Col1a1* and *Col1a2*) in the space of Disse cause sinusoidal capillarization, altering the fenestrated liver sinusoidal endothelial cell (LSEC) phenotype to a more defined vascular basement membrane [29,30]. The transformation of the sinusoids interferes with the molecular exchange between sinusoidal blood and hepatocytes, thereby compromising liver metabolism [29,30]. By secreting pro-fibrotic cytokines, aHSCs promote fibrosis generation, and, in turn, interaction with the fibrotic tissue activates HSCs [31]. Moreover, aHSCs suppress the resolution of the fibrotic ECM through changes in matrix metalloproteinase activity and the upregulation of the tissue inhibitors of metalloproteinase levels [32]. In this way, the activation of HSCs and the subsequent deposition of a fibrotic ECM creates a positive feedback loop, in which HSCs maintain a perpetually active state as chronic injury progresses [14] (Figure 2). Recently, single-cell RNA-sequencing revealed the distinct spatial zonation of HSCs, which can be designated as portal vein- or central vein-associated HSCs characterized by a high expression of nerve growth factor and ADAMTS-like 2 (a disintegrin and metalloproteinase with thrombospondin), respectively [33]. Central vein-associated HSCs were found to be the dominant source of collagen in CCl_4_-induced centrilobular fibrosis, and targeting these cells inhibited hepatic fibrosis [33]. As NASH is often characterized by centrilobular fibrosis, the zonation of HSCs and ability to target central vein-associated HSCs may have important consequences for the future development of precision medicine. Despite the initial centrilobular injury, NASH eventually involves most of the liver parenchyma, cholangiocytes, and hepatic progenitor cells that also play important roles in HSC activation. Chronic lipotoxic liver injury leads to hepatocyte senescence, which promotes cholangiocyte/progenitor cell proliferation and forms the so-called ductular reaction [5,34]. The reactive ducts secrete a range of pro-fibrotic factors (e.g., platelet-derived growth factor (PDGF) and transforming growth factor beta (TGFβ)) and correlate with fibrosis severity [5,35]. Consequently, blocking cholangiocyte secretin-signaling was found to reduce liver fibrosis by decreasing TGFβ-signaling [36]. This underscores the complexity of the cellular networks and crosstalk involved in HSCs in NASH.

Once injury ceases, fibrosis may resolve. Fibrosis regression is facilitated by ECM remodeling to remove scarring and re-establish a functional liver structure, and it requires a decrease in aHSCs [37]. During fibrosis regression, aHSCs are cleared through apoptosis or by becoming inactivated (iHSCs), reverting to a quiescent-like phenotype with a distinguishable gene expression profile more similar to qHSCs than aHSCs and with a lower threshold for re-activation in vivo [38,39] (Figure 2).

## 3. Mechanisms of HSC Activation

### 3.1. Lipotoxicity and Inflammation

**Figure 3:**
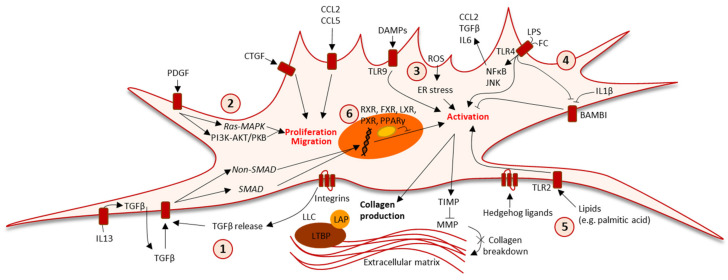
Molecular mechanisms of hepatic stellate cell activation. The activation of hepatic stellate cells involves multiple signaling pathways and receptor systems. 1: TGFβ is one of the most potent fibrogenic factors and is released in response to insults. In HSCs, TGFβ is released through IL-13-dependent induction and via integrin-mediated interactions with extracellular TGFβ stored in a LLC. TGFβ acts through SMAD and non-SMAD pathways to increase collagen synthesis and extracellular matrix deposition. An increased TIMP level inhibits MMP expression and collagen breakdown. 2: PDGF induces RAS-MAPK and PI3K-AKT/PKB signaling that—alongside cytokines and growth factors such as CCL2, CCL5, and CTGF—promotes HSC proliferation and migration. 3: Increased ROS induce ER stress, which (alongside DAMPs) leads to HSC activation. 4: Gut permeability may increase in NASH, and gut-derived and hepatic FC signaling through TLR4 promotes the production of inflammatory cytokines, growth factors, and HSC activation. In addition, TLR4 signaling can indirectly activate HSCs by decreasing the expression of the TGFβ decoy receptor BAMBI, which is also decreased by the inflammatory cytokine IL-1β. 5: In turn, lipotoxic lipid (e.g., palmitic acid) signaling through TLR2 and Hedgehog-derived signaling further contributes to HSC activation. 6: Nuclear receptors also play an important role in HSC activation, being inhibited by RXR, FXR, LXR, PXR, and PPARγ (decreased in activated HSCs). Though all mechanisms of HSC activation remain to be disclosed, this figure illustrates the highly complex cellular signaling patterns involved in NASH-associated HSC activation and the subsequent production of a fibrous extracellular matrix. AKT/PKB: protein kinase B. CTGF: connective tissue growth factor. BAMBI: bone morphogenetic protein and activin membrane-bound inhibitor. CCL: chemokine C-C motif ligand. DAMP: damage-associated molecular patterns. ER: endoplasmic reticulum. FC: free cholesterol. FXR: farnesoid X receptor. HSC: hepatic stellate cell. IL: interleukin. LPS: lipopolysaccharide. LAP: latency-associated protein. LLC: large latent complex. LTBP: latent TGF-β-binding protein. LXR: liver X receptor. MAPK: mitogen-activated protein kinase. MMP: matrix metalloproteinase. NAFLD: non-alcoholic fatty liver disease. PDGF: platelet-derived growth factor. PI3K: phosphoinositide 3-kinase. PPARγ: peroxisome proliferator-activated receptor γ. PXR: pregnane X receptor. ROS: reactive oxygen species. RXR: retinoid X receptor. TIMP: tissue inhibitor of matrix metalloproteinase. TGFβ: tissue growth factor β. TLR: toll-like receptor. SMAD: mothers against decapentaplegic homolog. Arrow heads indicate activation, and transversal lines indicate inhibition.

The excess lipid and cholesterol accumulation in hepatocytes can cause lipotoxicity by generating free radicals, such as reactive oxygen species (ROS), thereby promoting oxidative stress, compromising cellular metabolism and membrane integrity, and leading to decreased organelle function (e.g., mitochondrial dysfunction and endoplasmic reticulum (ER) stress) and the release of pro-inflammatory cytokines [2]. Hepatic cholesterol accumulation can activate HSCs directly by stimulating toll-like receptor 4 signaling or indirectly through an uptake of Kupffer cells that subsequently activate HSCs by secreting interleukin IL-1β, tumor necrosis factor (TNF), and TGFβ [40,41] (Figure 3).

The inflammatory response that is induced in NASH causes circulating monocytes to migrate to the liver, where they—together with the liver resident Kupffer cells—contribute to HSC activation and fibrosis by producing cytokines such as TGFβ, PDGF, TNF, interleukins, and chemokines [14]. TNF and IL-1β promote the survival of aHSCs through the activation of the NFκB pathway [42]. IL-1β exerts its pro-fibrotic function by upregulating tissue inhibitors of metalloproteinase 1 (encoded by *Timp1*) and downregulating bone morphogenetic proteins and activin membrane-bound inhibitors (a pseudoreceptor for TGFβ) in HSCs. In NASH patients, HSCs have been shown to express high levels of the IL-13 receptor, and IL-13 was shown to induce TGFβ and connective tissue growth factor (CTGF, which is encoded by *Ccn2*) production in HSCs in vitro [43,44]. Inflammatory chemokines aid HSC activation, and the deletion of chemokine (C-C motif) ligands CCL3 or CCL5 in mice administered CCl_4_ or a methionine/choline-deficient diet decreased HSC activation, hepatic fibrosis, and immune cell infiltration [45,46]. HSCs also express inflammation-inducing toll-like receptors, inducing activation in response to damage-associated molecular patterns released by compromised hepatocytes and ligands such as free fatty acids, lipopolysaccharide, and other microbial products that show elevated serum levels in NAFLD patients due to increased intestinal permeability and dysbiosis [47,48,49] (Figure 3).

### 3.2. Growth Factors

Hepatic TGFβ mRNA and serum TGFβ levels are increased in NASH patients, but a correlation to fibrosis grade is currently disputed [50,51]. TGFβ1 activation and signaling is induced in response to hepatocellular damage and ROS production, and it is a primary driver of HSC activation [52,53] (Figure 3). TGFβ is produced by several cell types including aHSCs, and stimulates HSC activation through the mothers against decapentaplegic homolog (SMAD) proteins SMAD2, SMAD3, and SMAD4, in turn inducing type I and III collagen expression and mitogen-activated protein kinase pathways [54,55,56,57,58,59]. In contrast, TGFβ induces SMAD7 in qHSCs, which inhibits the production of collagen I and III. This signaling-limiting regulation is absent in aHSCs, thus resulting in permanent TGFβ-mediated activation [60,61]. In vivo, the inhibition of TGFβ signaling was found to reduce HSC activation in a murine NASH model [62]. Latent TGFβ is stored in the ECM and can be activated through aHSC contraction mediated by integrins (a family of transmembrane receptors expressed by HSCs), subsequently promoting fibrogenesis [63] (Figure 3). Integrins also induce HSC activation through mechanosensing pathways in response to changes in ECM composition, thus enhancing fibrosis and placing integrins as key factors in the propagation of disease [31,64]. This role has been confirmed in vivo, where the inhibition of integrins or downstream mechanotransducers reduced CCl_4_-induced hepatic fibrosis in mice [64,65,66].

CTGF is a central mediator of TGFβ-dependent fibrogenesis. Expression has been found to be elevated in liver biopsies from NASH patients and serum levels have been found to be positively correlated with fibrosis stage in NAFLD patients, thus underlining a key role in disease and potential application as biomarker [67,68,69]. CTGF is induced by IL-13, supporting a link between chronic inflammatory signaling and the promotion of fibrosis that is possibly independent of TGFβ-induced signaling [44,70]. CTGF signaling upregulates cellular proliferation and survival, and it promotes the cellular ECM production, migration, and adhesion that are pivotal for aHSCs (Figure 3) [71]. Accordingly, CTGF overexpression was found to induce HSC activation in vivo, whereas its knockdown was found to inhibit aHSCs in vitro and to prevent CCl_4_-induced fibrosis in vivo [70,72]. 

PDGF signaling is also linked to HSC activation (Figure 3). The main active isoform PDGFB is produced by aHSCs and infiltrating macrophages, and the overexpression of PDGFB in mice has been found to induce HSC activation and liver fibrosis [73,74]. A central role of PDGF is supported by increased *PDGFRA* and *PDGFD* levels in NALFD patients with severe (F3–4) compared to mild (F0–1) fibrosis [75]. Moreover, the hepatic expression of platelet-derived growth factor receptor-beta (PDGFRβ) was found to be positively correlated with fibrosis severity in NAFLD patients [76]. PDGFRβ (encoded by *Pdgfrb*) is expressed by aHSCs but not qHSCs [77]. The auto-activation of PDGFRβ in HSCs from CCl_4_-treated or bile duct-ligated mice was found to accelerate fibrosis, whereas its depletion was found to decrease injury and fibrosis in vivo, supporting a key role in fibrogenesis [78]. 

PDGF also induces the phosphoinositide 3-kinase/protein kinase B-mediated production of Hedgehog (Hh) ligands in HSCs, while TGFβ and lipotoxicity stimulate Hh ligand secretion by hepatocytes [79,80,81]. Hh ligand binding in HSCs induces their activation and proliferation while inhibiting apoptosis, making the Hh pathway an important regulator of inflammation and fibrogenesis [82,83,84] (Figure 3). In NASH patients, Hh activity correlates with aHSC numbers and liver damage severity [85,86,87]. Inhibiting Hh signaling in Western diet-fed mice with NASH was found to improve fibrosis and hepatic inflammation, supporting a specific role of the Hh pathway in NASH-related fibrosis [88]. Hh signaling might also influence HSC activation by inducing the expression of genes involved in glycolysis and lactate accumulation. This metabolic switch is thought to facilitate the altered gene expression profile of aHSCs and is linked to hypoxia-inducible factor-1 alpha expression [89]. The centrilobular distribution of NASH-associated fibrosis is in line with the reduced oxygen tension across the liver-lobule towards the central vein, and it is accompanied by an increased expression of hypoxia-inducible factor-1 alpha in NASH patients [90,91]. A study in high fat fed mice further indicated a profibrotic role for hypoxia-inducible factor-1 alpha, warranting the future exploration of the effect of hypoxia on HSC fate [92].

### 3.3. Nuclear Receptors

Nuclear receptors such as retinoic acid receptors, liver X receptors, peroxisome proliferator-activated receptors (PPARs), farnesoid X receptors (FXRs), and pregnane X receptors form heterodimers with the retinoid X receptor and modulate gene expression in response to dietary ligands such as cholesterol, fatty acids, and bile acids, all of which are linked to cholesterol metabolism and NAFLD [93,94]. Liver X receptors are nuclear cholesterol sensors, and liver X receptor alpha positively regulates sterol regulatory element binding protein, which is highly expressed in qHSCs and downregulated during HSC activation [95]. Sterol regulatory element binding protein inhibition was found to increase type I collagen expression in cultured HSCs, whereas liver X receptor ligands were found to suppress HSC activation in vitro [96,97]. HSC-specific *PPARγ* deletion was shown to aggravate hepatic fibrosis, while *PPARγ* overexpression decreased HSC activation and fibrosis in vivo [98,99]. FXR expression is decreased in NASH patients and inversely correlated with NAFLD activity score [100]. FXR agonists have been found to upregulate PPARγ expression and to decrease activation markers in HSCs in vitro, as well as to reduce hepatic fibrosis in vivo [101,102,103]. Conversely, high fat fed *LDLr-/-/FXR-/-* mice were shown to have increased hepatic inflammation and collagen deposition [104]. Polymorphisms of the pregnane X receptor, which is regulated by FXR, have been linked to increased disease severity in NAFLD patients [105,106]. Pregnane X receptor agonism inhibited HSC activation in vitro and CCl_4_-induced liver fibrosis in vivo [107,108] (Figure 3).

### 3.4. Cellular Stress and Autophagy

Increased cellular stress and free radical production play pivotal roles in NAFLD-induced inflammation, TGFβ activation, and fibrogenesis [53]. Accordingly, antioxidant supplementation (caffeic acid phenethyl ester, sestrin 2, and curcumin) has been shown to decrease HSC activation in vitro and to prevent or ameliorate hepatic fibrosis in rodent models, supporting antioxidants as beneficial in the prevention and potential resolution of disease [109,110,111,112]. 

Reactive oxidant species also promote ER stress in HSCs, which, in turn, stimulates autophagy and HSC activation, and proteins associated with ER stress and autophagy are commonly dysregulated in NAFLD patients [113,114] (Figure 3). Inhibiting autophagy has been found to attenuate HSC activation and proliferation in vitro, as well as to reduce fibrosis in thioacetamide- or CCl_4_-treated mice [115,116]. Autophagy also plays a role in HSC activation because the activated cells decrease their stored retinoid droplets [117,118]. However, genetically modified mice incapable of storing retinoids in HSCs showed no difference in fibrosis severity in response to bile duct ligation or CCl_4_ treatment [119]. In contrast, the application of retinoids suppressed HSC activation in vitro and reduced fibrosis in CCl_4_-treated animal models [120,121,122]. Thus, the significance of HSC retinoid autophagy is still unclear. Conversely, ER stress may also increase aHSC clearance by increasing apoptosis and, in turn, reducing fibrogenesis, suggesting differential effects of induced ER stress in HSCs [123].

## 4. HSC Inactivation and Apoptosis

While HSC activation pathways have been extensively studied in vitro and in models of fibrotic diseases, the role of HSC inactivation and its potential value as a pharmacological target have not been explored to the same degree. 

The expression of the characteristic qHSC marker PPARγ is abolished during HSC activation, but the stimulation of PPARγ can halt aHSC proliferation, induce apoptosis, or reverse aHSCs to quiescent-like iHSCs, and it has been shown to ameliorate liver fibrosis in vivo [99,124,125,126]. HSC-specific PPARγ knockout (*Pparg-/-*) in mice was shown to not only exacerbate fibrosis development in response to CCl_4_ but also slow fibrosis regression after the cessation of treatment accompanied by the persistent expression of *Col1a1*, *Acta2*, and αSMA, thus indicating continued HSC activation [27,98]. The PPARγ agonist rosiglitazone accelerated fibrosis resolution in wildtype mice after the termination of CCl_4_ administration and coincided with lower levels of *Col1a1*, *Timp1*, *Acta2*, and αSMA, as well as upregulation of *Pparg* compared to recovering vehicle treated mice [27]. These findings indicated a specific role for PPARγ in HSC inactivation and its importance for fibrosis resolution. 

HSCs alter their gene expression profile during activation, which is accompanied by a change in transcription factor expression. Transcription factor 21, involved in fetal HSC differentiation, is decreased in cultured aHSCs and in fibrotic human and murine liver tissue, but it is increased after the discontinuation of CCl_4_ treatment in mice coinciding with fibrosis regression [127,128]. The overexpression of transcription factor 21 was found to upregulate qHSC marker genes (*Gfap* and *Ngfr*) and to downregulate profibrotic genes (*Pdgfrb*, *Acta2*, and *Col1a1*) in vitro, and it was found to further reduce *Acta2* and *Col1a1* expression in mice with CCl_4_– or methionine/choline-deficient diet-induced liver fibrosis, accompanied by the regression of fibrosis and steatohepatitis [128]. However, PPARγ expression or lipid droplet uptake were not restored, indicating that complete HSC inactivation was not achieved [128].

Human aHSCs were inactivated in vitro by stimulation with a cocktail containing growth factors, palmitic acid, and retinol, thus leading to the downregulated expression of αSMA and type 1 collagen, as well as the reduction of proliferation and matrix metalloproteinase activity [129]. ECM organization and retinol metabolism were partly restored to levels exhibited by qHSCs, and 70% of cells accumulated cytoplasmatic lipid droplets, underlining a switch in phenotype [129]. While most gene expression markers were similar to those of in vivo generated iHSCs, PPARγ expression was not restored in vitro [38,129]. The application of retinol and palmitate alone was also shown to induce HSC inactivation in vitro, as indicated by decreased αSMA and collagen type I expression and an increased lipid droplet storage [130]. However, since saturated free fatty acids like palmitic acid promote NAFLD, the translational potential of this findings remains to be assessed [47,48].

During capillarization, LSECs lose the ability to prevent HSC activation through vascular endothelial growth factor A-stimulated nitric oxide synthesis, but they might actively stimulate HSC activation by secreting proinflammatory cytokines [29,131,132]. Conversely, the co-culturing of aHSCs with differentiated LSECs resulted in HSC inactivation, as measured by a reduced expression of αSMA and collagen type I, as well as the re-establishment of cytosolic fat droplets [29]. The pharmacological stimulation of nitric oxide production in rats with thioacetamide-induced liver cirrhosis restored the differentiated LSEC phenotype, which subsequently led to the apoptosis and inactivation of aHSCs [133]. While studies have shown lower vascular endothelial growth factor A levels in NASH patients compared to healthy controls or to patients with bland steatosis, hepatic angiogenesis driven by vascular endothelial growth factor A is thought to aid fibrogenesis; therefore, possible interventions targeting LSEC-mediated HSC inactivation should concentrate on downstream effectors [134,135,136]. 

Extracellular vesicles can alter the phenotype of their recipient cells and may prove a novel approach to NASH treatment [137]. Accordingly, extracellular vesicles from qHSCs reversed the phenotype of activated HSCs by transferring *Ccn2*-inhibiting miRNAs, which were diminished in aHSCs in vivo after thioacetic acid or CCl_4_ treatment [138]. Extracellular vesicles derived from healthy primary murine hepatocytes or AML12 (alpha mouse liver) cells induced the downregulation of *Acta2*, *Ccn2*, and *Col1a1* expression in aHSCs in vitro [139]. Similarly, serum-derived extracellular vesicles from healthy mice suppressed fibrogenesis and decreased aHSC markers in CCl_4_-treated mice [140]. Likewise, extracellular vesicles from healthy human subjects decreased human hepatic stellate cell line LX-2 activation [140]. This supports extracellular vesicles as important signaling molecules in the reversion of HSC activation and the putative resolution of NASH. 

In summary, the above findings reflect the complexity of factors influencing HSC inactivation. One major challenge is the determination and evaluation of the inactivation status, since not all quiescence markers and morphological characteristics may be regained by iHSCs, while some activation markers remain.

Additionally, apoptosis clears aHSCs from the liver, thereby restoring it by removing the primary source of fibrogenic matrix production and increasing matrix resolution, e.g., by reducing aHSC-induced tissue inhibitor of metalloproteinase 1 (TIMP1) expression. Accordingly, aHSC apoptosis has been shown to reverse CCl_4_-induced hepatic fibrosis in vivo [32]. In pursuing this strategy, several pathways have been suggested as potential targets. This includes the inhibition of NFκB-dependent gene transcription by sulfasalazine, promoting the apoptosis of αSMA-positive stellate cells, and reducing collagen 1 and TIMP1 production, thus leading to the reversion of hepatic fibrosis in vivo [141]. In mice, aHSC apoptosis was achieved by inhibiting C/EBP-α (member of the CCATT/enhancer binding protein family), ultimately promoting the resolution of CCl_4_-induced hepatic fibrosis [142]. Cultured primary human HSC (αSMA-positive) showed the expression of TNF-related apoptosis inducing ligand (TRAIL) receptors, with the subsequent blocking of TRAIL-R3 and R4 leading to an increased susceptibility to killing by natural killer cells and suggesting TRAIL-mediated regulation as important in the clearance of aHSCs [143]. However, a limitation in the application of apoptosis-promoting agents is a lack of efficiency in targeting specific cell populations, consequently leading to serious side effects. Cell-penetrating peptides specific for aHSC internalization and subsequent intracellular drug release have been shown to effectively target aHSC in vitro and lead to apoptosis due to cargo-mediated induction [144]. This may prove valuable in the development of novel approaches to fibrosis resolution though aHSC apoptosis.

## 5. Pharmacotherapies with Putative Effects on HSCs

Several of the compounds currently undergoing clinical evaluation may affect fibrosis through HSC activation or/and inactivation (Table 1). With cenicriviroc, the application of CCLR2 and 5 dual antagonists as putative treatment for NASH-associated liver fibrosis, entered phase III clinical trial after showing fibrosis improvement without worsening of NASH in phase II, however the study was recently terminated due to a lack of efficacy (trial id: NCT03028740) [145,146,147]. Suggested mechanisms include a direct effect on HSC activation by C-C chemokine receptor type 5 antagonism and an indirect effect by inhibiting the recruitment of circulating monocytes (C-C chemokine receptor type 2-mediated), as indicated by increased hepatic levels of anti-inflammatory macrophages and decreased pro-inflammatory macrophages in a diet-induced NASH mouse model after cenicriviroc treatment [148]. As detailed above, cellular stress and the ensuing apoptosis contribute to the activation of HSC and the progression of NASH. Apoptosis signal-regulating kinase 1 mediates apoptosis induced by ROS, inflammation, and ER stress, thus constituting an attractive therapeutic target [149]. However, the apoptosis signal-regulating kinase 1 inhibitor selonsertib was not found to improve fibrosis or facilitate NASH resolution in NASH patients with bridging (F3) fibrosis (6 mg, *n* = 321; 18 mg, *n* = 322) or cirrhosis (F4) (6 mg, *n* = 351; 18 mg, *n* = 354) compared to placebo (*n* = 159 and *n* = 172, respectively) (NCT03053050 and NCT03053063) [150].

Though PPARs have been proposed to modulate HSC activation, clinical findings have yet to confirm their effects on NASH-mediated fibrosis. PPARγ agonist pioglitazone showed an improvement of NASH endpoints (steatosis, inflammation, and ballooning hepatocytes) but did not significantly improve fibrosis regression in patients with impaired glucose tolerance/type 2 diabetes (45 mg/day, *n* = 26) or non-diabetic patients with NASH (30 mg/day, *n* = 70) compared to placebo controls (*n* = 21 and *n* = 72, respectively) [151,152]. Moreover, elafibranor, a dual PPARα and PPARβ/δ agonist, failed to significantly improve NASH and fibrosis in a phase III clinical trial (NCT02704403) [153]. Lanifibranor—a pan-agonist affecting PPARα, PPARγ and PPARβ/δ—is currently showing promising results, achieving NASH resolution and fibrosis regression in a phase IIb clinical trial (NCT03008070) [154].

The FXR agonist obeticholic acid is currently in a phase III clinical trial for NASH treatment (NCT02548351) after two different phase II studies in NAFLD or NASH patients indicated a positive effect on fibrosis (NCT00501592 and NCT01265498) [155,156]. The planned interim analysis confirmed significant improvements in the fibrosis of at least one stage without the worsening of NASH, which was achieved by 23% of patients with stage F2 or F3 fibrosis treated with 25 mg of obeticholic acid (*n* = 308) compared to 12% in the placebo group (*n* = 311), but these patients also encountered adverse effects such as pruritus (47 (7%) in the placebo group, 109 (17%) in the 10 mg of obeticholic acid group, and 115 (17%) in the 25 mg of obeticholic acid group) and elevation of low density lipoprotein (123 (19%) in the placebo group, 183 (28%) in the 10 mg of obeticholic acid group, and 336 (51%) in the 25 mg of obeticholic acid group) [157]. Consequently, approval based on these findings was not granted by the FDA (Food and Drug Administration, USA). The included examples of prospective treatment options support effects in NASH, and several showed a beneficial effect on NASH-associated hepatic fibrosis. Collectively, putative effects on HSC activation (either direct or indirectly) remain to be shown.

## 6. Conclusions

In ascertaining a pivotal role in NASH-induced hepatic fibrosis, HSCs and their activation/inactivation represent an interesting therapeutic target. While markers of HSC activation are becoming increasingly known, the inactivated phenotype is less understood. The current incomplete insight into the regulatory mechanisms of the qHSC–aHSC–iHSC interplay in NASH restricts our understanding of the signaling pathways of disease-associated fibrosis and concurrent resolution. The further exploration of HSCs and the mechanisms driving the phenotypic switch in NASH is therefore necessary if efforts to identify potential HSC targets for drug development are to succeed.

## Figures and Tables

**Table 1:** Clinical trials of pharmacotherapies to improve NASH-associated liver fibrosis.

Drug	Mode of Action	Status	Outcome	Trial No.
Cenicriviroc	C-C chemokine receptor type 2 and 5 dual antagonist	Phase III trial terminated due to lack of efficacy in fibrosis improvement (primary endpoint)	No results available	NCT03028740
Selonsertib	Apoptosis signal-regulating kinase 1 inhibitor	Phase III trial terminated due to lack of efficacy in fibrosis improvement (primary endpoint)	≥1-stage fibrosis improvement without worsening of NASH in 9.6% (18 mg of drug), 12.1% (6 mg of drug) and 13.2% (placebo); *p*-value 0.49 (18 mg) and 0.93 (6 mg)	NCT03053050
		Phase III trial terminated due to lack of efficacy in fibrosis improvement (primary endpoint)	≥1-stage fibrosis improvement without worsening of NASH in 14.4% (18 mg of drug), 12.8% (6 mg of drug) and 12.8% (placebo); *p*-value 0.56 (18 mg) and 0.93 (6 mg)	NCT03053063
Pioglitazone	PPARγ agonist	No improvement of fibrosis regression in phase III trial (secondary endpoint)	Decrease in fibrosis score in 44.3% (drug) and 30.6% (placebo); *p*-value 0.12	NCT00063622
		No improvement of fibrosis regression in phase IV trial (secondary endpoint)	Decrease in fibrosis score in 46% (drug) and 33% (placebo); *p*-value 0.08	NCT00227110
Elafibranor	PPARα and PPARβ/δ dual agonist	Phase III trial did not meet the predefined primary efficacy endpoint of NASH resolution without fibrosis worsening ^1^	No significant difference in the improvement of fibrosis between treatment and placebo groups	NCT02704403
Lanifibranor	PPARα, PPARγ and PPARβ/δ pan-agonist	Phase IIb trial achieved NASH resolution and fibrosis regression (secondary endpoints)	≥1-stage fibrosis improvement without worsening of NASH in 42% (1200 mg of drug), 28% (800 mg of drug) and 24% (placebo) of ITT population; *p*-value 0.011 (1200 mg) and 0.53 (800 mg) Resolution of NASH and fibrosis improvement in 31% (1200 mg of drug), 21% (800 mg of drug) and 7% (placebo) of ITT population; *p*-value <0.001 (1200 mg) and 0.017 (800 mg)	NCT03008070
Obeticholic acid	FXR agonist	Ongoing phase III trial, fibrosis improvement at planned interim analysis (primary endpoint) ^2^	≥1-stage fibrosis improvement without worsening of NASH in 23% (25 mg of drug), 18% (10 mg of drug) and 12% (placebo); *p*-value 0.0002 (25 mg) and 0.045 (10 mg)	NCT02548351

^1^ Favorable results were achieved in a post-hoc analysis with a modified definition. ^2^ Accelerated approval of obeticholic acid was not granted by the FDA (Food and Drug Administration, USA). Additional data are currently pending. ITT: Intention to treat.

## Data Availability

No new data were created or analyzed in this study. Data sharing is not applicable to this article.
